# Aging mind and brain: is implicit learning spared in healthy aging?

**DOI:** 10.3389/fpsyg.2013.00817

**Published:** 2013-11-07

**Authors:** James H. Howard, Darlene V. Howard

**Affiliations:** ^1^Department of Psychology, The Catholic University of AmericaWashington, DC, USA; ^2^Department of Psychology, Georgetown UniversityWashington, DC, USA

**Keywords:** aging, implicit learning, cognition, striatal dysfunction, cognitive neuroscience

## Abstract

It is often held that although explicit learning declines in the course of normal aging, implicit learning is relatively preserved. Here we summarize research from our group which leads us to argue that some forms of implicit learning do decline with adult age. In particular, we propose that there are age-related declines in implicit learning of probabilistic sequential relationships that occur across the adult lifespan, and that they reflect, at least in part, age-related striatal dysfunction. We first review behavioral evidence supporting this age-related decline and then evidence from patient groups, genetics, and neuroimaging supporting this striatal dysfunction hypothesis.

People are remarkably sensitive to spatial and temporal relationships among objects and events. These relationships are typically *probabilistic* and *sequential*, in that events often, but not always, predict later ones. Learning about these relationships is important throughout life because it enables people to perceive the world efficiently, to learn and use language, to make decisions based on experience, and even to engage in social interaction (Saffran et al., 1996; Lieberman, 2000; Kuhl, 2004; Conway and Pisoni, 2008). An intriguing characteristic of such *probabilistic sequence learning* is that it often takes place outside of awareness in that we need not make any special effort to learn, and are unable to articulate what we have learned. As a result of such learning, we know that a sentence is ungrammatical without being able to cite the syntactic rules it violates or we have a bad feeling about a new acquaintance without being able to express what social cues led to that impression. Reber (1967) termed this *implicit learning* to distinguish it from deliberate, effortful explicit/declarative learning.

It is often claimed, particularly in general reviews of cognitive aging, that implicit, unlike explicit, learning and memory are age invariant. But there is a wide range of implicit processes, and different forms of implicit learning. It appears that some of these *are* spared with aging, including repetition priming as well as some skills learned early and then practiced throughout life (Ikier et al., 2008; Morrow et al., 2008; Laver, 2009). Nonetheless, none of these involves picking up subtle, sequential probabilistic regularities. In fact, one skill that does call, at least in part, on implicit learning, second-language acquisition (Hedenius et al., 2011; Morgan-Short et al., 2012a,b; Frost et al., 2013), apparently declines in a linear fashion with adult age (Hakuta et al., 2003).

This perspective paper does not offer a review of the growing literature on implicit learning and aging (see review by King et al., 2013). Instead, we aim to summarize findings from our own group, including our students and collaborators, supporting two claims. First, we propose that *implicit probabilistic sequence learning is not spared in healthy aging*; rather there are persistent age-related learning deficits that can be detected as early as middle age. Second, we propose that these declines reflect changes in the brain that occur with aging, specifically, *declines in a striatal-based learning system*.

## Age-related differences in learning

Most studies of implicit learning and aging have examined perceptual/motor based sequence learning, using the Serial Response Time (SRT) task (Nissen and Bullemer, 1987). In the original SRT people respond as quickly as possible to each of a series of events that, unbeknownst to them, follow a regular repeating spatial pattern. With practice, people become faster at responding overall, reflecting general improvement. More importantly, sequence-specific learning is revealed by the fact that response times increase abruptly if the sequential regularity is removed. Virtually all SRT studies find that older adults do show sequence-specific learning, but studies differ in whether age deficits are detected. The reason for the different results is unclear. Most studies revealing age equality in learning have used relatively simple deterministic regularities, in that each event or sequence of events perfectly predicts a subsequent one (Howard and Howard, 1989, 1992; Frensch and Miner, 1994; Cherry and Stadler, 1995; Salthouse et al., 1999; Dennis et al., 2006; Gaillard et al., 2009).

**Figure 1:**
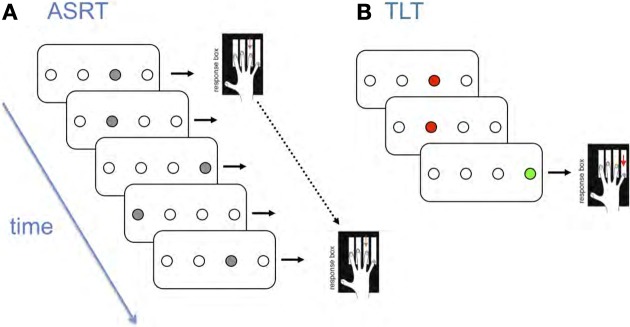
**Schematic representation of (A) the Alternating Serial Reaction Time Task (ASRT) and (B) The Triplets Learning Task (TLT)**.

In contrast, our work using probabilistic versions of the SRT consistently reveals age-related deficits. For example, as illustrated in Figure 1A, we developed an Alternating Serial Response Time (ASRT) task, which differs from the traditional SRT in that predictable and random events occur on alternate trials, thereby introducing probabilistic 2nd order structure (i.e., event *t* predicts event *t* + 2 with high probability). For example, a given participant might encounter the regularity 1r2r4r3r1r2r4r3r …., where 1–4 represent different events and *r* represents any one of these. Thus, some runs of three events or *triplets*, occur with high probability because they conform to the regularity (e.g., 1×2 here where × is any event), whereas others occur infrequently (e.g., 2×1), thereby manipulating the conditional probabilities of successive events. Our findings suggest that people do not learn the alternating regularity *per se*, but rather become sensitive to the predictive relationships (Howard and Howard, 1997); with practice, RT and accuracy for these two triplet types diverge, with increasingly faster and more accurate responding to high relative to low probability triplets.

We have reported that young, middle-aged and old adults do learn 2nd order probabilistic sequences in the ASRT, but there are age deficits especially as training progresses (Howard and Howard, 1997; Howard et al., 2004). We have also shown that age-related deficits occur for spatial and non-spatial stimuli, persisting even with extended practice distributed over as much as 10 h of training (Dennis et al., 2003, 2006; Negash et al., 2003; Howard et al., 2004). In fact, what is most striking is that while old adults learn as much as young early in training, they asymptote well below younger people.

These age deficits in learning are detectable in middle-age (aged 34–53) (Feeney et al., 2002) and are more pronounced in healthy old-old than young-old adults (aged 65–73 and 76–80, respectively) (Howard and Howard, 1997), suggesting there might be a gradual decline in learning across the adult lifespan. Janacsek et al. (2012) have replicated adult age deficits in ASRT learning using a lifespan sample, but longitudinal data are needed. Age deficits are even more pronounced when a 3rd-order regularity is used (i.e., event *t* predicts event *t* + 3), and under some conditions older adults do not learn 3rd-order associations even with extended training (Howard et al., 2004; Bennett et al., 2007).

To investigate whether the age deficits in the ASRT are due to older adults' difficulties with motor sequencing, we developed a Triplets Learning Task (TLT, Figure 1B). It is similar to the ASRT, but rather than responding to every item in a stream, participants observe two red cues and then respond to a third green target on each of a series of three-event trials (Howard et al., 2008). We then vary the relation between the cue and target events; for example, for 2nd-order regularities (comparable to the ASRT) the first cue probabilistically predicts the target, and for 1^st^-order regularities the second cue predicts the target. The TLT yields learning data that are very similar to the ASRT; young and old adults learn equally well early in training, but old adults asymptote at a lower level (Howard et al., 2008; Simon et al., 2010). These TLT findings indicate that the age-related deficits in ASRT learning are not due solely to motor sequencing deficits.

Age deficits appear in the TLT for 1st as well as for 2nd order regularities (Howard et al., 2008; Stillman et al., 2012), suggesting that even simple first-order probabilistic structure can lead to age differences. This is important, because given the slower processing and reduced capacity that accompanies aging (Salthouse, 1996), deficits in 2nd and higher-order learning might reflect a reduced availability of the events to be associated, rather than a learning deficit *per se*. The fact that older adults are poorer than young at learning 1st order structure, where only immediately adjacent events need to be associated, suggests a deficit in associative learning, and also indicates that age related deficits are not limited to learning higher order structure.

There is always concern that observed age deficits might reflect explicit “contamination” such that the young are better, not because of superior implicit learning, but because they have gained more explicit/declarative knowledge than older people. However, the age deficits observed in the ASRT and TLT are unlikely due to age differences in explicit knowledge for several reasons. First, sensitive tests indicate people are not aware of the regularity. For example, people are unable to differentiate between high and low probability triplets in a subsequent recognition test even with substantial learning during the ASRT and TLT tasks (Howard et al., 2004, 2008). In addition, using an “implicit/explicit” variation of the ASRT that enables people to gain explicit knowledge, we found that implicit learning occurs independent of any explicit knowledge that is acquired (Song et al., 2007). There is also evidence that learning in the TLT, unlike explicit learning, is not capacity demanding; both old and young people learn 2nd order regularities even in the presence of a working memory load, with little or no effect on learning (Gamble et al., in press). Finally, both old and young adults show near-perfect retention of an ASRT sequence over at least 1 year, consistent with the excellent long-term retention characteristic of implicit/procedural skills (Romano et al., 2010).

We have focused here on our own group's work using the ASRT and TLT, but we are not alone in finding age-related deficits in implicit sequence learning. For example, Curran (1997) found age deficits when 12-element 2nd-order deterministic SRT sequences were intermixed with blocks of random sequences whereas Daselaar et al. (2003) found age equivalence using a similar procedure. Maddox and colleagues (Filoteo and Maddox, 2004; Maddox et al., 2010) reported age deficits in information-integration learning on a classification task known to rely on the striatal system. Further, Weiermann (Weiermann and Meier, 2012) has reported age deficits in a study requiring simultaneous learning of both a repeating task and response sequence.

In summary, there is accumulating evidence that not all forms of implicit learning are spared the negative effects of aging, suggesting that there is *a fundamental deficit in the system underlying learning of probabilistic sequential regularities*. Some of our more recent work investigates the neural bases of these age differences.

## Biological foundations of age differences in learning

Recent research indicates that in young adults, implicit sequence learning involves two interacting neural systems; one based primarily on striatal (including caudate) and the other on medial temporal lobe (MTL, including hippocampus) networks (Henke, 2010; Dennis and Cabeza, 2011). The MTL system is characterized by the relatively fast acquisition of associations, and predominates early in training, whereas the striatal system learns more slowly, gradually building up associations among events (Poldrack and Packard, 2003) and becoming increasingly important as training progresses.

There is cross-sectional and longitudinal evidence that the striatum undergoes substantial age related declines in structure and function beginning in early adulthood, including declines in the overall level of striatal dopamine and in dopamine receptor density (Raz et al., 2005; Backman et al., 2010). In contrast, recent research suggests that the MTL is relatively spared in healthy aging, at least until very advanced age, even though it is affected early in Alzheimer's disease (Hedden and Gabrieli, 2005; Raz et al., 2005).

Thus, our recent work has been guided by a *striatal aging hypothesis* which proposes that age-related deficits in implicit probabilistic sequence learning reflect declines in a striatal-based learning system (Howard and Howard, 2012), similar to proposals advanced by others (Filoteo and Maddox, 2004; Poldrack and Foerde, 2008; Rieckmann and Bäckman, 2009). This hypothesis is consistent with the pattern of age deficits in the TLT and ASRT described above. This includes the evidence suggesting a gradual decline in learning across the adult years, similar to the gradual decline in the striatum, and the finding that age deficits are greater late in practice, when the striatum is known to dominate learning. To obtain more direct evidence, we have used patient and genetics studies as well as structural and functional neuroimaging with healthy young and older adults as illustrated below.

Consistent with the *striatal aging hypothesis*, we have demonstrated selective impairment of ASRT learning in individuals with corticobasal syndrome (CBS), a progressive neurological disorder characterized by atrophy of striatal regions with symptomatology similar to Parkinson's Disease (Negash et al., 2007). We have also shown that TLT learning is related to the dopamine transporter gene, DAT1, which plays an important role in striatal dopamine signaling. A variant of this gene, the 9-repeat allele, is associated with higher synaptic dopamine levels and with greater caudate volume and activity (Bertolino et al., 2006). We observed more learning in college-aged 9-carriers compared to 10/10 homozygotes on the TLT (Simon et al., 2011). Furthermore, this advantage only emerged late in training when responding would be expected to depend largely on the striatal system for young people. Taken together, these data suggest the importance of striatal dopamine for learning in the ASRT and TLT.

We have used fMRI to demonstrate that striatal activation is related to implicit sequence learning in the TLT for both young and older adults (Simon et al., 2012). Simon contrasted activation to high vs. low probability target events, i.e., a “response to predictability,” and found that early in training, younger adults displayed a greater caudate response than older adults. Furthermore, in late training, the degree of learning correlated positively with caudate response across young, but not older adults, suggesting that late in training young adults relied more on striatal networks than did old. In contrast, the degree of late learning correlated positively with hippocampal activation (response to predictability) for older adults, but not younger. Taken together, these findings suggest that the balance between the two neural systems differs in young and older adults. This is consistent with the *striatal aging hypothesis* and with recent evidence that in the face of this striatal deficit, older adults rely to a greater degree on the MTL system for implicit learning (Rieckmann and Bäckman, 2009; Dennis and Cabeza, 2011; Simon et al., 2012).

Although most neuroimaging research has focused on age-related changes in the structure and function of gray matter, there are also widespread declines in the integrity of white matter (Sullivan and Pfefferbaum, 2007; Madden et al., 2009; Bennett et al., 2010). We have used diffusion tensor imaging (DTI) tractography to show that the integrity of white matter tracts in cortico-striatal networks relates to age-deficits in the ASRT thereby further implicating these networks in implicit learning (Bennett et al., 2011). Bennett assessed the integrity of several tracts including a caudate-dorsolateral prefrontal cortex (DLPFC) tract in both young and older adults, using a measure of fractional anisotropy (FA) such that higher FA indicates high white matter integrity. Consistent with the *striatal aging hypothesis*, older adults showed lower FA for the caudate-DLPFC tracts than younger adults, whereas FA did not differ between groups for the other tracts we examined (including a hippocampal-DLPFC tract). More importantly, individuals with greater caudate-DLPFC tract FA revealed more learning. This positive correlation was observed for both young and older adults, but with older adults showing lower FA and less learning overall than young. In addition, left caudate-DPLFC tract integrity mediated the age differences in late learning, with tract FA attenuating the age group effect on learning by 93%. These findings complement studies of gray matter substrates and suggest that age-related white matter loss in striatal tracts contributes to age deficits in learning.

## Summary and future directions

The above studies show that there are age deficits in implicit probabilistic sequence learning, and they point to striatal aging as a contributing factor. However, important questions remain. As we indicated above, the characteristics of an implicit learning task that yield age differences remain unclear, and the exact conditions under which the striatum is important for learning are not fully known (see, e.g., Schultz, 2007; Gallistel and Matzel, 2013). We have argued here that age deficits are observed consistently with *probabilistic* sequences, whether simple first-order or more complex higher-order structure, because such sequences are optimally learned via the striatal system. However, to the extent the striatum is needed for optimal learning of other relationships, including perhaps complex deterministic ones, age deficits will also be observed.

In addition, we focused here on age *group* differences, but there are substantial differences in sequence learning among individuals of the same age (e.g., Janacsek et al., 2012). Recent studies have begun to identify the source of these differences, including the evidence above that within-group sequence learning is related to dopamine genotype (Simon et al., 2011) and to white matter integrity (Bennett et al., 2011). We have also found relationships with life experience, including musical training and videogaming (Romano-Bergstrom et al., 2011).

Additional work should also address the implications of these learning differences for domains such as reading, decision-making, second language learning, and social cognition. For example, ASRT and TLT learning are associated with reading ability as assessed with standardized diagnostic tests in young (Howard et al., 2006) and older adults (Bennett et al., 2008) and may contribute to adult age differences in learning a second language (Schwab et al., 2013). Age-deficits in experience-based decision making may also reflect a learning impairment (Mata et al., 2011; Seaman et al., 2013).

Beyond this, additional work should investigate the neural dynamics that accompany implicit learning. We have recently used functional connectivity methods to show that intrinsic connectivity between the caudate and MTL is correlated with sequence learning in young adults (Stillman et al., in press), and we have used EEG/ERP to examine within-trial processes (Kiser et al., 2012).

To summarize, throughout life people are able to learn implicitly about regularities in their environment, enabling them to adapt to a changing world. Although some kinds of implicit learning are spared, we have reported evidence that implicit probabilistic sequence learning is *not* spared, likely reflecting age-related changes to the striatal system. Future work should further investigate the role of the striatum in individual differences in brain/behavior relationships as well as the implications for a broad range of important everyday tasks.

## Author contributions

The authors have contributed equally to the work.

### Conflict of interest statement

The authors declare that the research was conducted in the absence of any commercial or financial relationships that could be construed as a potential conflict of interest.
